# A feasibility and safety study of concurrent chemotherapy based on genetic testing in patients with high-risk salivary gland tumors: Preliminary results

**DOI:** 10.1097/MD.0000000000010564

**Published:** 2018-04-27

**Authors:** Rongrong Li, Shengjin Dou, Min Ruan, Chenping Zhang, Guopei Zhu

**Affiliations:** Department of Oral and Maxillofacial-Head Neck Oncology, Ninth People's Hospital, Shanghai Jiao Tong University, School of Medicine, Shanghai, P.R. China.

**Keywords:** concurrent chemotherapy, drug sensitivity, genetic testing, head and neck cancer, salivary gland tumor, tailored therapy

## Abstract

**Background:**

This prospective study was conducted to evaluate the feasibility and safety of customized chemotherapy regimens based on the gene characteristics of salivary gland tumors.

**Methods:**

Patients were enrolled with histologically confirmed intermediate or high grade, stage T3–4, N1–3 disease, and T1–2, N0 patients with a close (≤1 mm) or microscopically positive surgical margin were also enrolled in the study. All patients received radical surgery and postoperative concurrent chemoradiotherapy. To evaluate the responsiveness of therapies, the chemotherapy regimen was based on gene targets, β-tubulin III, ABCB1, STMN1, and CYP1B1 (for docetaxel) and TYMS (for pemetrexed). The primary endpoints were treatment compliance and acute toxicities.

**Results:**

A total of 20 patients were enrolled between September 2013 and January 2016. The median age was 46 years (range: 23–70 years). Genetic testing showed that 8 patients may have been sensitive to docetaxel, 5 patients may have been sensitive to pemetrexed, and 7 patients sensitive to either docetaxel or pemetrexed. All patients received the full dose of radiation. A total of 19 patients (95%) completed 2 cycles of concurrent chemotherapy (CCT). One patient treated concurrently with pemetrexed experienced grade 3 neutropenia. Three patients experienced grade 3 oral mucositis, and 2 patients experienced grade 3 dermatitis.

**Conclusion:**

Our study demonstrated that a CCT selecting method based on the gene targets associated with drug sensitivity was clinically feasible and safe. Further studies enrolled more patients with longer follow-up times are needed to confirm the clinical efficacy of this CCT selecting method.

## 1 Introduction

Salivary gland tumors (SGTs) are rare, heterogeneous groups of tumors that comprise less than 5% of head and neck cancers and takes approximately 0.5% of all malignancies.^[1]^ They vary considerably in their phenotypic, biological, and clinical behaviors, as well as in prognosis. Postoperative radiotherapy is generally advocated in cases of adverse prognostic factors undifferentiated and high-grade tumors, advanced disease, close or positive margins, and perineural invasion. Although no randomized controlled trials were conducted, numbers of institutional experiences suggested a remarkable improvement in local control and overall survival (OS) time with surgery followed by postoperative radiotherapy compared to surgery alone.^[2–4]^ However, local failure rates still approached 20%. The rates of distant metastases are approximately 20% depending on histology and grade.^[5]^ Although concurrent chemotherapy (CCT) and radiation have achieved notable success in more common squamous cell head and neck squamous cell carcinomas, it is unknown yet if additional chemotherapy beyond radiation is better than radiation alone in SGTs. Platinum-based concurrent chemoradiotherapy (CCRT) is mostly adopted to locally advanced (stage III/IV) head and neck squamous cell carcinomas. However, a standard chemotherapy regimen for SGTs is not available owing to the rarity and histologic heterogeneity.

In the past decades, tailored therapy has made unprecedented progress in various cancers. Some genetic markers in tumor samples have been found to be associated with the response to chemotherapy. They have the potential to guide the selection of chemotherapy regimen. In this study, we chose several genetic markers to evaluate drug responsiveness in SGT and predict the therapy efficacy of CCT in SGT. The genetic chosen markers were β-tubulin III, ABCB1, STMN1, and CYP1B1 for docetaxel and TYMS for pemetrexed. They all have been well established predicting response in other cancers.^[6–11]^ The selected patients were treated with postoperative radiation with CCT based on these genetic markers. This is a prospective study performed to evaluate the feasibility and safety of customized CCT regimens based on the genetic markers of SGTs.

## 2 Material and methods

### 2.1 Patient selection

This was a nonrandomized, phase II trial. In this study, patients were enrolled in Shanghai Ninth People's Hospital, Shanghai Jiaotong University School of Medicine. Patients were eligible if they had histologically confirmed intermediate or high grade SGTs, stage T3–4, N1–3, a close surgical margin (≤1 mm), or microscopically positive surgical margins. The 7th AJCC/UICC staging system was used. Other inclusion criteria included an age of 18 to 70 years and a Karnofsky performance status of at least 70%. Adequate hematologic, hepatic, and renal functions were also required. Exclusion criteria were as follows: distant metastases, another noncured cancer except for basocellular carcinoma of skin, and prior history of radio(chemo)therapy treatment to head and neck region. Informed consent was obtained from all individual participants included in the study. The study was approved by local independent ethics committee. All patients had radical surgery followed by postoperative radiotherapy.

### 2.2 Postoperative radiotherapy

Prior to treatment, patients were immobilized in a supine position with a custom-made head/neck/shoulder mask. CT simulation with 5-mm thick slices was performed. Gross target volume was not recorded because all patients had surgical resection of the gross tumor. The clinical target volumes were defined for the surgical/tumor bed, possible invasive regions, and subclinical microscopic disease. The planning target volumes were created by expansion of 5 mm beyond clinical target volumes. The target delineation was in accordance with the protocol of RTOG 1008.

Patients were treated with 3-dimensional conformal radiotherapy or intensity modulated radiotherapy with daily fraction of 1.8 to 2.0 Gy, 5 fractions administered per week. According to our protocol, patients with stage I or stage II cancers received 60-Gy to primary tumor bed and 54-Gy to ipsilateral upper neck (level Ib and II). Patients exhibiting certain risk factors (stage III/IV cancer, extracapsular extensions, perineural invasion, and/or positive margins) received 66-Gy to primary tumor bed and 54-Gy to upper neck (level Ib and II) for N0 cases, whereas comprehensive ipsilateral nodal irradiation (level Ib to V) was only applied to N+ cases. The contralateral neck was excluded from the radiation field except for midline primary lesions or primary lesions within 1 cm of the midline.

### 2.3 Concurrent chemotherapy guided by genetic testing

The chemotherapy regimen was determined according to the results of genetic testing. When the results showed that neither docetaxel nor pemetrexed was sensitive to the patients, cisplatin was adopted. We analyzed the β-tubulin III, STMN1, and TYMS protein expression status and the genotype of TYMS, ABCB1 2677 G>T/A, and CYP1B1 Leu432Val polymorphisms. The combined results predict the drug responsiveness. These targets involve in different pathways of drug absorption, transportation, metabolism, etc., which may possibly affect the therapy efficacy.

Formalin-fixed paraffin-embedded surgical tumors and normal tissues were used for testing. DNA was extracted using the QIAamp DNA FFPE Tissue Kit (QIAGEN, Germany), and blood DNA was extracted using TIANamp Genomic DNA Kit (TIANGEN, Beijing, China). For each patient, the tumor mRNA levels of STMN1, TUBB3, and TYMS genes were measured by fluorescent real-time polymerase chain reaction. Predetermined values for these genes, which were generated from large cohorts of Chinese patients, were used to dichotomize expression levels following the manufacturer's instructions. The TYMS genotyping was performed in normal and tumor tissues. Genotyping of TYMS gene can be affected by the loss of heterozygosity on 18p in tumor DNA. The tumor TYMS genotyping was evaluated by knowing the allelic status of the tumors. The allele frequencies of MDR-1 SNP G2677T/A and CYP1B1 SNP Leu432val were genotyped as described by Gréen et al^[6]^ and Bailey et al, respectively.^[12]^ The chemotherapy regimen was determined according to the sensitivity results shown in Table 1.

**Table 1:**
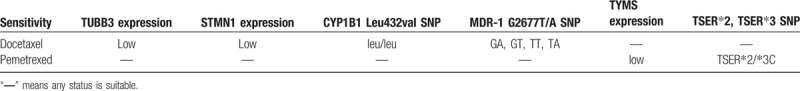
Sensitivity results determined by gene targets.

All patients received CCT according to the sensitivity results. The following regimens were used in individual patient depending on the sensitivity test. Each patient planned to undergo at least 2 cycles of chemotherapy.

Docetaxel only: 80 mg/m^2^ on day 1, every 21 days.

Pemetrexed only: 500 mg/m^2^ on day 1, every 21 days.

Cisplatin only: 70 mg/m^2^ on day 1 to day 3, every 21 days.

### 2.4 Evaluation during and after treatment

Patients were evaluated weekly during radiotherapy, then every 3 months for the first 2 years and every 6 months thereafter. Acute and late toxicities (defined as beyond 3 months of completion of treatment) were recorded according to the Common Terminology Criteria for Adverse Events v3.0 (CTCAE v3.0). Physical examination, MRI or CT for head and neck, chest CT, and abdominal ultrasound were performed at each follow-up visit.

### 2.5 Statistical analysis

The primary endpoints were treatment compliance and acute toxicities. The study treatment was considered feasible if the withdrawal rate from CCRT due to toxicity was less than 10%. The secondary endpoints of this study were local recurrence-free survival (LRFS), regional recurrence-free survival, distant metastasis-free survival (DMFS), and OS. Follow-up time was calculated from the date of treatment initiation to the date of the last contact or death. Time to failure was calculated from the date of treatment initiation to the date of the relevant event. Survival analyses were computed using the Kaplan–Meier method.

## 3 Results

### 3.1 Patient characteristics

Between September 2013 and January 2016, a total of 20 patients were included in the trial. The median age was 46 years (range: 23–70 years); 8 patients (40%) were male; and 12 patients (60%) were female. The most common histologic type of cancer was mucoepidermoid carcinoma, which occurred in 8 patients (40%). Stage distributions were as follows: stage II, 3 patients; stage III, 8 patients; stage IVa, 7 patients; and stage IVb, 2 patients.

The clinical characteristics are listed in Table 2.

**Table 2:**
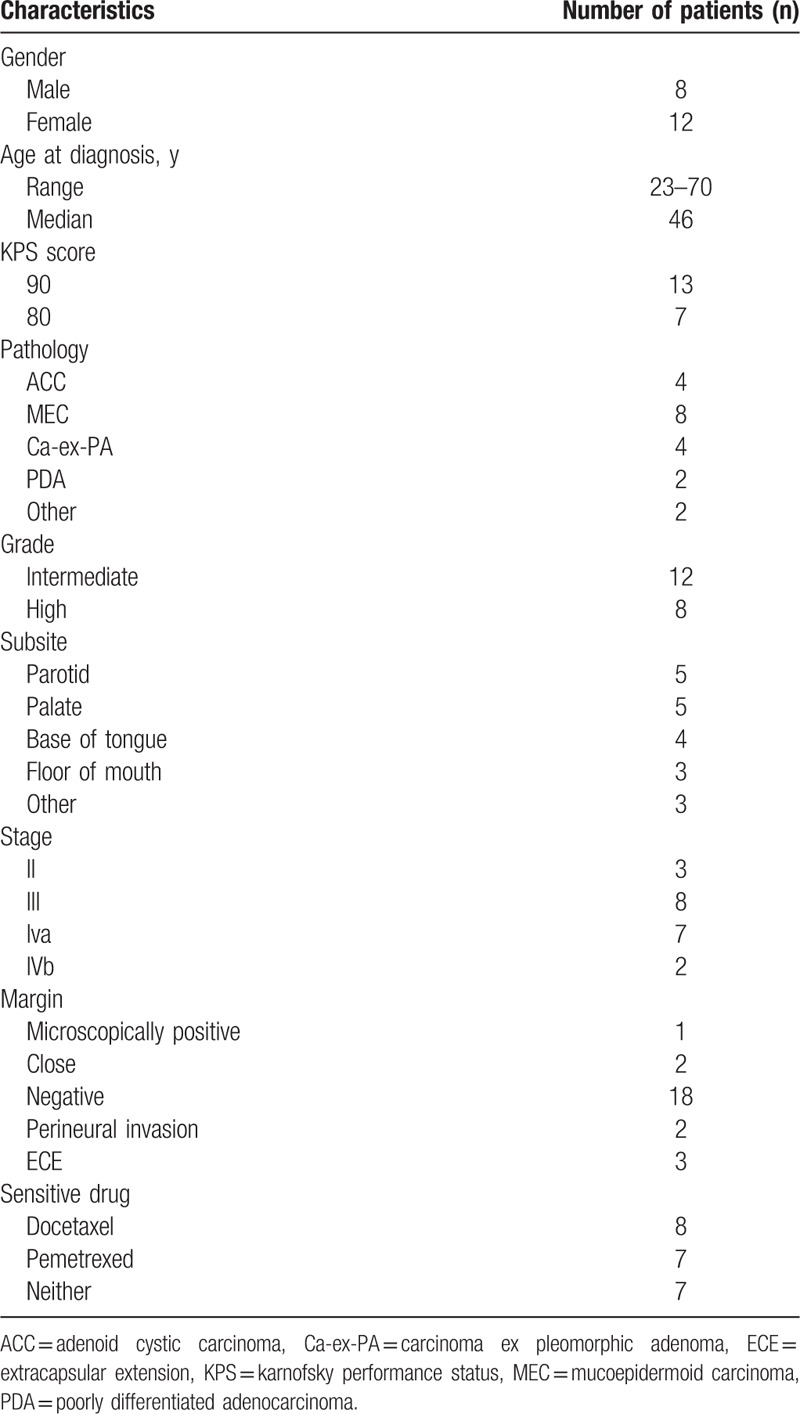
Patient and disease characteristics.

### 3.2 The genetic characteristics and chemotherapy

Table 3 summarized the genetic characteristics of all patients. The results showed that 8 patients may be sensitive to docetaxel, and 2 of 8 patients may be sensitive to both docetaxel and pemetrexed. These patients received CCT with docetaxel. Five patients may be sensitive to pemetrexed only, and they were treated with pemetrexed. The remaining 7 patients may not be sensitive to either docetaxel or pemetrexed received CCT with cisplatin (Table 3).

**Table 3:**
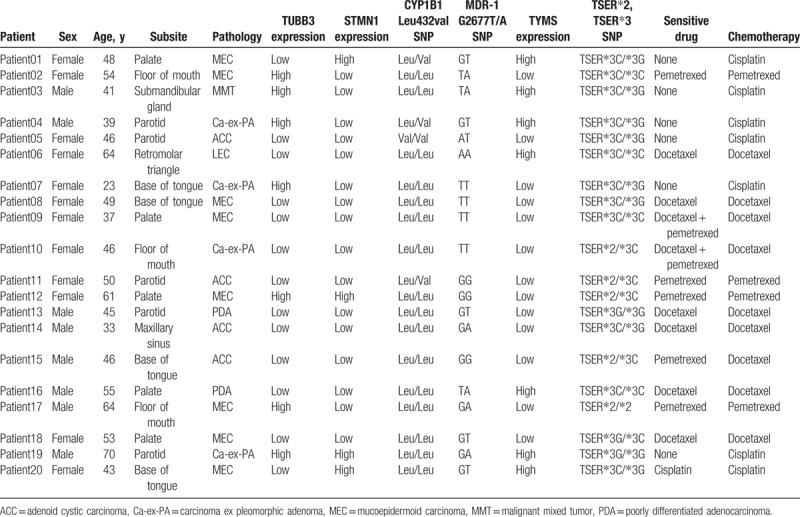
The genetic testing results of all patients and chemotherapy drugs received.

### 3.3 Survival analysis

**Figure 1:**
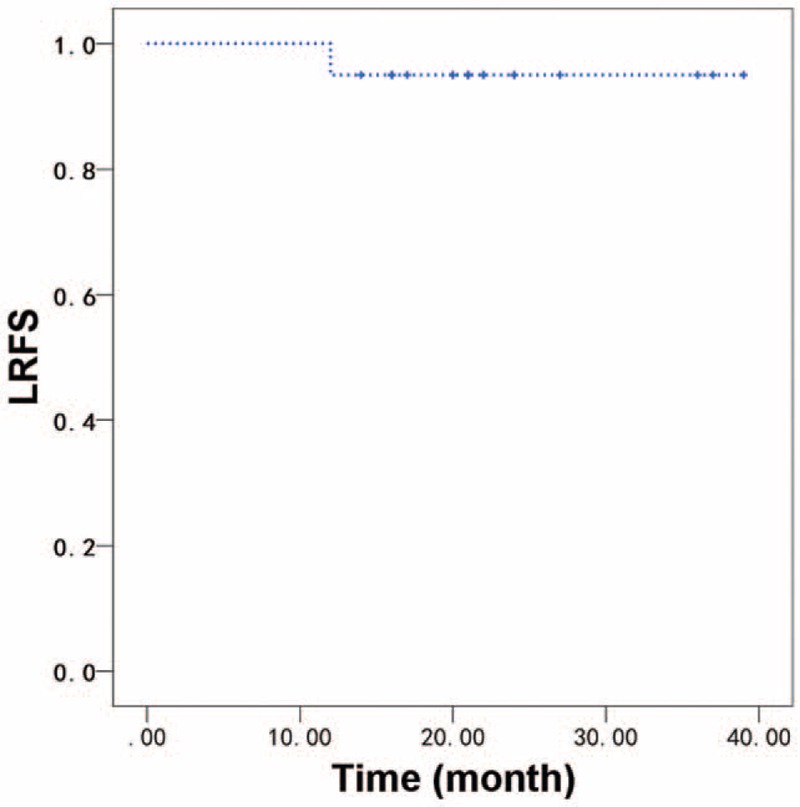
LRFS of all patients received customized chemotherapy regimens based on the gene characteristics. Two-year LRFS was 87.5%. LRFS = local recurrence-free survival.

**Figure 2:**
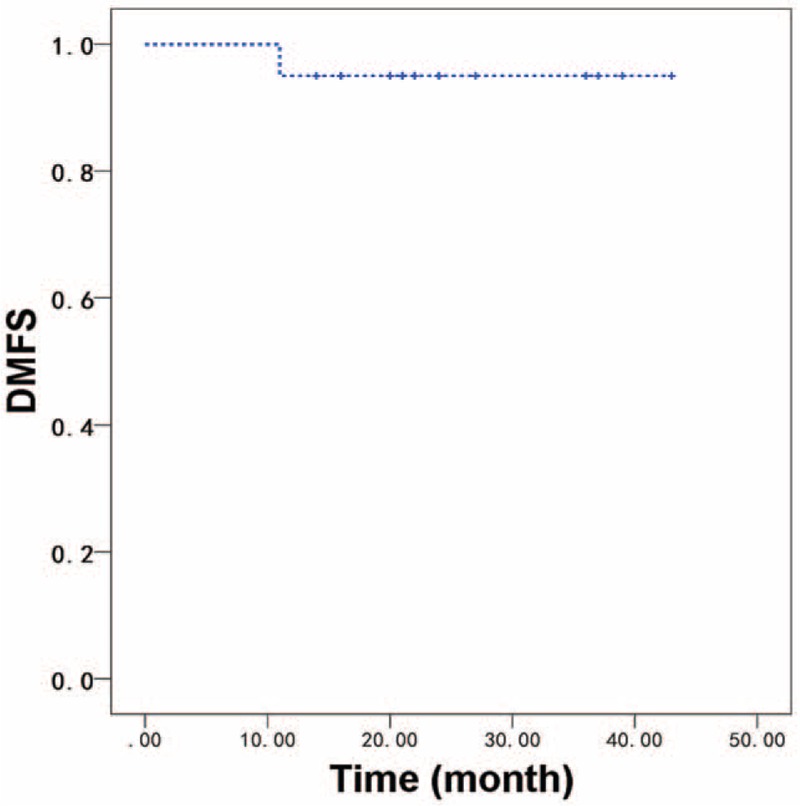
DMFS of all patients received customized chemotherapy regimens based on the gene characteristics. Two-year DMFS was 95.0%. DMFS = distant metastasis-free survival.

The median follow-up time for all patients was 21 months (range: 14–43 months). One patient developed local recurrence 12 months after radiotherapy. This patient had stage III (T3N1M0) high grade MEC of the base of the tongue and received definitive CCRT with cisplatin. One patients developed lung metastasis 11 months after radiotherapy. This patient had stage III (T3N0M0) ACC of parotid gland. There was no regional recurrence. No treatment related death was reported. For all patients, the 2-year OS, LRFS, regional recurrence-free survival, and DMFS were 100%, 87.5%, 100%, and 95.0%, respectively (Figs. 1 and 2).

### 3.4 Treatment compliance

All patients received full dose of radiation. Nineteen patients (95%) completed all 2 cycles of CCT. One patient treated with pemetrexed discontinued the planned CCT because of grade III neutropenia after the 1st cycle of CCT. Three patients experienced treatment delays, 1 due to grade III oral mucositis while 2 due to machine breakdown. The duration of treatment delays was 1, 2, and 2 days, respectively.

### 3.5 Acute toxicity

No treatment-related deaths occurred, and no patient experienced grade 4 toxicity during CCRT. Almost all patients experienced mild and moderate acute toxicities. It included grade 0–2 oral mucositis in 17 patients, neutropenia in 19 patients, dermatitis in 18 patients, xerostomia in 20 patients, vomiting in 20 patients, and dysphagia in 20 patients. Severe toxicities (grade 3 or above) were infrequent. One patient treated with concurrent pemetrexed experienced grade 3 neutropenia. However, the neutrophil count recovered to grade I after 1 week following the administration of granulocyte colony-stimulating factor. This patient received only 1 cycle of CCT. No patients had febrile neutropenia or infection related to the treatment. Three patients with oral cavity SGTs experienced grade 3 oral mucositis. Two patients experienced grade 3 dermatitis. Renal function impairment and ALT/AST elevation was not found in the patient cohort. The detailed acute toxicities are listed in Table 4.

**Table 4:**
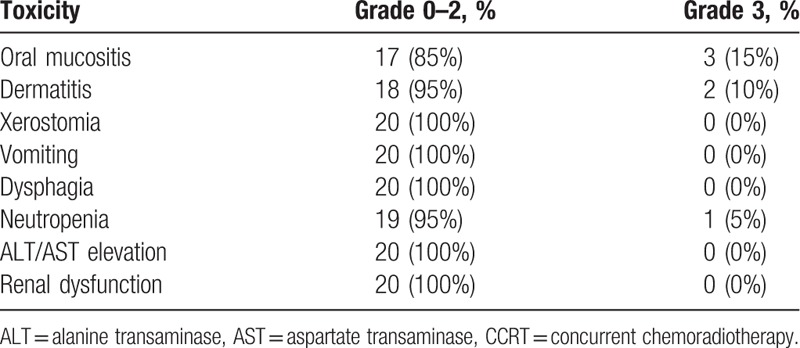
Incidence of acute toxicities during CCRT.

## 4 Discussion

Due to the extreme rarity and heterogeneity of SGTs, the role of CCT with radiation in the management of SGTs is not clear yet. Some retrospective studies have shown that CCRT has achieved excellent rates of local control for patients but along with multiple unfavorable disease characteristics.^[5,13]^ Cisplatin is the most common chemotherapy agent using in the combination with radiation therapy. Currently, an ongoing RTOG clinical trial, RTOG 1008, examines the role of addition of weekly cisplatin treatment to adjuvant radiation in high risk SGTs. This was initially a phase II study, now expanded to phase III study, comparing adjuvant concurrent radiation and cisplatin treatment versus radiation alone in resected high-risk malignant SGTs. The results will not be available in next couple of years.

Because of the heterogeneity and diversity of SGTs, a tailored chemotherapy regimen may be desirable in individual patients depending on the sensitivity of the tumor to selected chemotherapeutic agents. Biological factors have been considered (expression of p53, c-ERB2, EGFR, MUC, and c-kit, etc.) to guide a systemic approach. However, reliable long-term results are not available yet, and preliminary results did not support these markers to be predictors.^[14–16]^ We then evaluated a strategy to tailor chemotherapy based on the expression level of the genes associated with drug sensitivity.

A growing body data suggested that several genetic markers can predict outcome patients treated with chemotherapy. High expression of class III β-tubulin has been associated with either low response rates to taxane or vinorelbine-containing regimens.^[11]^ Cancer patients who are homozygously mutated for the missense mdr-1 SNP, G2677T/A, respond better to treatment with taxane than those with at least 1 wild-type allele^[6,17]^; CYP1B1–4326C>G (Leu432Val) polymorphism emerged as possible predictive marker of response and clinical outcome to docetaxel^[10,18]^; TYMS overexpression in tumor cells correlated with reduced response to pemetrexed-containing chemotherapy might be a predictor of sensitivity to pemtrexed-based chemotherapy^[19,20]^; the effectiveness of pemetrexed monotherapy also depends on polymorphisms in TS gene,^[21,22]^ thus, TS gene polymorphisms could be accounted as molecular predictor factors for pemetrexed-based chemotherapy. As docetaxel and pemetrexed are common agents currently being used in the adenocarcinomas treatment, few studies took them concurrently with postoperative radiotherapy in the SGTs treatment. We intended to use both drug instead of cisplatin under the guidance of genetic testing to achieve better outcomes.

However, chemotherapy activity was varied, the response rates of cisplatin were modest, survival advantages were still unclear.^[23]^

This study showed that postoperative radiotherapy with CCT based on genetic testing is a feasible and safe treatment strategy in patients with high-risk SGTs. The toxicity was manageable while did not lead to a delay of radiotherapy. The treatment compliance observed in this study was favorable compared to the compliance observed in head and neck cancers.^[24]^ Importantly, comparing to the commonly adopted in hospital chemotherapy in China, the CCT regimen administered in the outpatient clinic, is both patient-friendly, logistically attractive, and cost effective. Postoperative radiotherapy combined with CCT was well tolerated, with a modest expected increase in acute toxicity rates occurred, most notably in grade 2 and grade 3 mucositis and dermatitis. Acute grade 4 or grade 5 toxicity was not observed. These results were comparable with the aforementioned results in the retrospective studies.^[13,25]^ Therefore, CCT seems to have minimal impact on morbidity and mortality associated with postoperative radiotherapy, the 2-year OS, LRFS, and DMFS of the patient cohort were 100%, 87.5%, and 95.0%, respectively.

To the best of our knowledge, this study was the first study designed to test feasibility and safety of tailored chemotherapy based on genetic testing in the SGTs treatment. There are limitations in our study. The correlations between drug sensitivity and genetic targets were frequent in other tumors but head and neck. The sample size was small and the follow-up time was short. Nevertheless, our findings are worthy for further investigation in a randomized trial with more patients and longer follow-up.

## 5 Conclusions

Our study demonstrated a CCT selecting method based on the gene targets associated with drug sensitivity is clinically feasible and safe. Further prospective studies enroll more patients with longer follow-up times are needed to confirm the clinical efficacy of this CCT selecting method. Although no definitive conclusion can be determined that this method benefits patients and results in better survival rates. Currently, our results demonstrated that this method was well tolerated. Considering the potential benefit of this method, tailored CCT is one of the most important avenues for personalized medicine in the treatment of SGTs. Prospective long-term studies are needed.

## Acknowledgments

The authors thank Shanghai Municipal Commission of Health and Family Planning (grant number 201640158) for the support.

## Author contributions

**Conceptualization:** Rongrong Li, GUOPEI ZHU.

**Data curation:** Shengjin Dou.

**Formal analysis:** Shengjin Dou.

**Funding acquisition:** GUOPEI ZHU.

**Investigation:** Rongrong Li.

**Methodology:** Shengjin Dou.

**Project administration:** Rongrong Li.

**Resources:** Rongrong Li, Min Ruan, GUOPEI ZHU.

**Software:** Shengjin Dou.

**Supervision:** Min Ruan, Chenping Zhang, GUOPEI ZHU.

**Validation:** Rongrong Li, Min Ruan, Chenping Zhang.

**Visualization:** Min Ruan, Chenping Zhang.

**Writing – original draft:** Shengjin Dou.

**Writing – review & editing:** GUOPEI ZHU.
